# Deleting *fis* downregulates virulence and effectively protects *Pasteurella multocida* infection in mice

**DOI:** 10.1186/s12917-025-04769-x

**Published:** 2025-05-07

**Authors:** Zhijie Wang, Siyu Liu, Muhan Xie, Zhengchun Lang, Xuan Zhang, Liang Luo, Guangfu Zhao, Nengzhang Li, Yuanyi Peng

**Affiliations:** College of Veterinary Medicine, Southwest University, Chongqing, 400715 China

**Keywords:** *Pasteurella multocida*, *Fis* gene, Capsule, Iron utilization, Virulence, Vaccine, Cross protection

## Abstract

*Pasteurella multocida* (*P. multocida*) is an important pathogen causing various diseases in both domestic and wild animals. The factor for inversion stimulation (Fis) is a nucleoid-associated protein with diverse functions in various bacteria, which positively regulate the transcription of capsular glycosaminoglycan genes in *P. multocida*. However, the precise mechanistic understanding of how the *fis* regulate virulence of *P. multocida* remains largely unknown. In this study, we discovered that *fis* transcript levels of *P. multocida* CQ2, serotype A (PmCQ2) were significantly increased in vivo, and showed a positive correlation with the capsule and virulence of *P. multocida*. To further understand how the *fis* regulated *P. multocida* pathogenesis, a homologous recombination strategy was used to generate *fis*-deleted strain. Then, the growth velocity, virulence characteristics, immune/inflammatory responses, and the survival rates of challenged mice were determined. The findings revealed that the presence of *fis* promoted the growth, regulated synthesis of capsule and biofilm of PmCQ2, and helped to resist phagocytosis by macrophages. Notably, we firstly demonstrated that Fis determined whether *P. multocida* can use bound iron ion for its survival. Consequently, the loss of *fis* greatly restricted *P. multocida* pathogenicity, as evidenced by reducing tissue bacterial loads as well as inflammatory factors levels. Moreover, the *fis* deletion strain was endowed with strong cross immunoprotected properties against *P. multocida* serotype A and B. Thus, these results suggested the pathogenic role of *fis* in *P. multocida* and proposed that *fis* deletion strain could be used as an attenuated vaccine candidate against *P. multocida* of serotype A and B.

**Supplementary Information:**

The online version contains supplementary material available at 10.1186/s12917-025-04769-x.

## Background

*P. multocida* is an important veterinary pathogen that can cause various animal diseases, including hemorrhagic sepsis in ungulates, avian cholera in poultry, and atrophic rhinitis in pigs [1]. Various environmental stresses in animals lead to an increase of pasteurellosis caused by *P. multocida*, which results in significant economic losses. *P. multocida* can be classified into five capsular serotypes (A, B, D, E, F) based on capsular polysaccharides [2–4]. Each capsular serotypes produces a distinct capsular polysaccharide. The capsule is an important virulence factor required by the bacteria to survive. Although multiple genes, including *phyA, phyB, hyaE, hyaD, hyaC, hyaB, hexD, hexC, hexB and hexA* are involved in capsule biosynthesis and transport [5, 6], the transcription of the *P. multocida* capsular biosynthesis loci is mainly manipulated by the factor for inversion stimulation (*fis*) [7, 8]. The Fis of *P. multocida* is a nucleoid-associated protein comprising 99 amino acid residues and containing a helix-turn-helix DNA binding motif in residues 73–94 that is responsible for interactions with capsular DNA promoter regions, which positively regulate the transcription of capsular glycosaminoglycan genes [9]. The function of Fis is also different in various bacteria. The Fis represses curli synthesis [10], activates *virF* [11], produces the plasmid-borne toxin *Pet* [12], and negatively regulates the phase variation *fimS* switch of the type 1 pilus operon [13] in pathogenic *Escherichia coli*. In *Pseudomonas aeruginosa*, Fis manipulates type III secretion system by influencing the transcription of *exsA* [14]. In addition, the functions of Fis have been revealed in many other bacteria, including *Salmonella enterica* [15–17], *Shigella flexneri* [18], *Vibrio cholera* [19] and so on. However, few studies focused on the *fis* gene of *P. multocida*. In our previous study, *fis* of *P. multocida* was highly expressed in vivo as detected by transcriptomics. Based on previous studies, we hypothesized that *fis* is an important virulence factor of *P. multocida*.

In this study, we collated previous studies about the gene deletion strain of *P. multocida* by our group and confirmed the positive correlation between capsule and virulence. We first demonstrated that *fis* manipulated the intake of bound iron ion by regulating haemoglobin and/or haemin receptors in *P. multocida*. Our data supported that the *fis* gene of *P. multocida* regulated a variety of biological functions and affected bacterial virulence, and mice vaccinated with the live deleted *fis* strain (live Δ*fis* strain) showed a strong cross-protection against *P. multocida* serotype A and serotype B.

## Results

The correlation between bacterial capsules and virulence and construction of the *fis* deletion strain, complementary strain and overexpress strain.

**Fig. 1:**
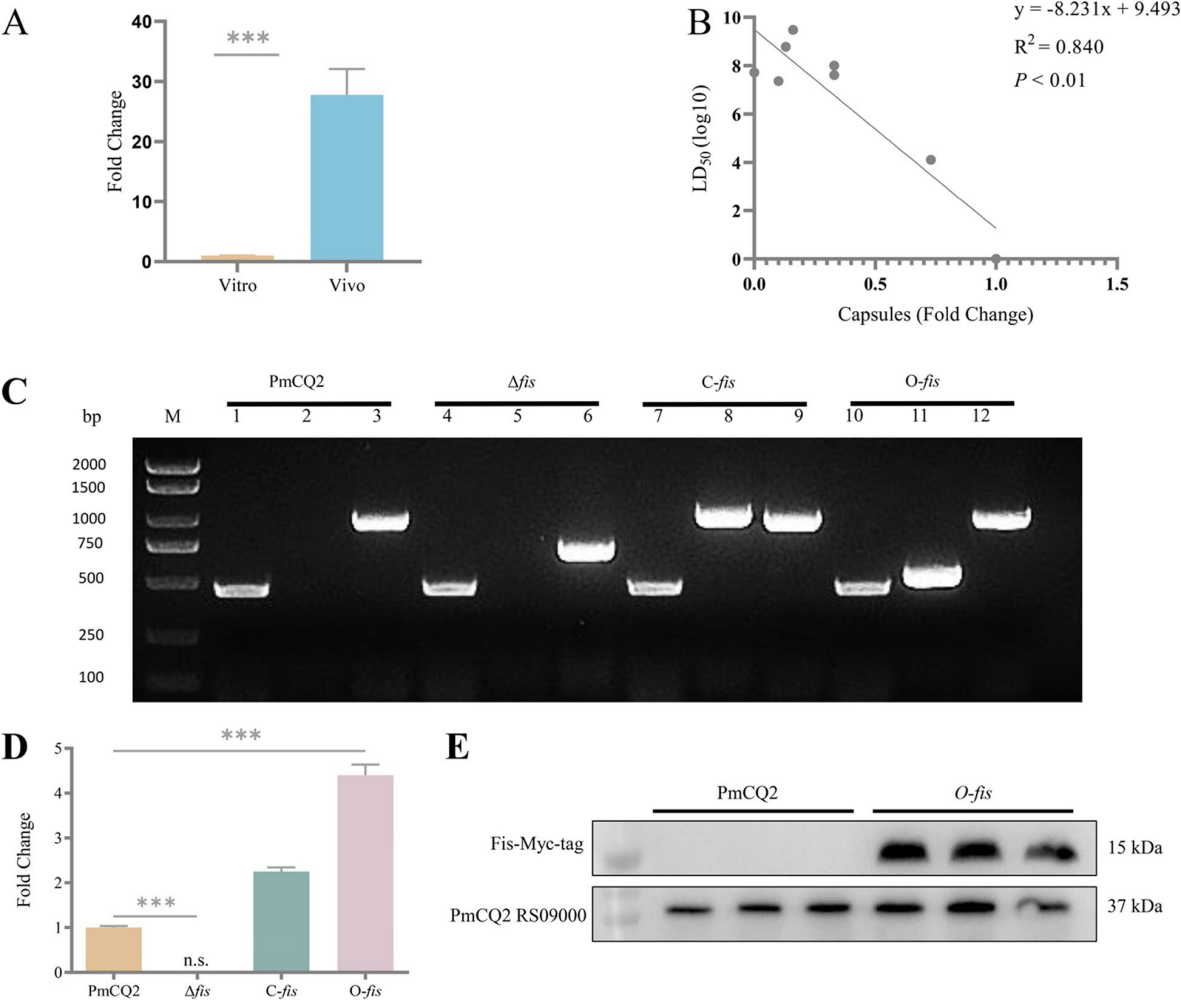
The correlation between bacterial capsules and virulence and construction of the *fis* deletion strain (Δ*fis*), complementary strain (C-*fis*) and overexpress strain (O-*fis*). A: Transcript levels of the *fis* gene (PmCQ2) in vitro and in vivo cultures. B: Correlation analysis between PmCQ2 capsule and LD_50_. C: PCR confirmation of PmCQ2, Δ*fis*, C-*fis* and O-*fis*. M: DNA marker; Lanes 1, 4, 7 and 10: *P. multocida* detection by using species-specific primers KMT1-F/KMT1-R; Lanes 2, 5, 8 and 11: Plasmids detection by using primers KO-F/KO-R and OE-F/ OE-R. Lanes 3, 6, 9 and 12: *fis* gene detection by using primers Fis-UUF/Fis-DDR. D: *fis* gene transcripts measured by using qRT-PCR. E: *fis* gene overexpression measured by using Western Blot (PmCQ2-RS09000 protein as an internal reference). All data are expressed as mean ± SD. * *P* < 0.05; ** *P* < 0.01; *** *P* < 0.001; ns means no significant

In our previous transcriptomic study, we found that *fis* transcript levels in PmCQ2-infected mice were 11.98-fold higher than that in Martin medium. Then, qRT-PCR were used for validation and showed that *fis* transcript levels of PmCQ2 were significantly higher in mice than in the Martin medium (Fig. 1A). We collated previous studies [20–24] about gene deletion strains (8 strains, including PmCQ2 and 2 non-publicly available strains) of *P. multocida* by our group, and analyzed the correlation between their capsules and Lethal Dose 50% (LD_50_), and drew regression lines. The results showed that there was a significant negative correlation between capsule and LD_50_ (*P* < 0.01) (Fig. 1B).

To investigate the role of Fis in *P. multocida*, we constructed a *fis* deletion strain (Δ*fis*), a *fis* overexpressed strain (O-*fis*), and a *fis* gene complementary strain (C-*fis*) derived from Δ*fis*. The construction process of plasmids and mutant strains is shown in Supplementary Fig. 1A and B. PCR validation showed that the *fis* gene was not in the chromosome of Δ*fis*, but existed in a plasmid and chromosome of C-*fis* and O-*fis* (Fig. 1C). To further confirm the *fis* deletion, complementary and overexpressed strains, RT-PCR and Western Blot were conducted, respectively. RT-PCR showed that the *fis* gene transcripts only existed in PmCQ2, C-*fis* and O-*fis*, but not in Δ*fis* (Fig. 1D). In addition, the *fis* gene transcription of O-*fis* was higher than that in other strains. Western Blot showed that the Fis with Myc-tag expressed in O-*fis* but not in the PmCQ2, suggesting the successful construction of O-*fis* strain (Fig. 1E).

### The characteristics of Δ*fis*

**Fig. 2:**
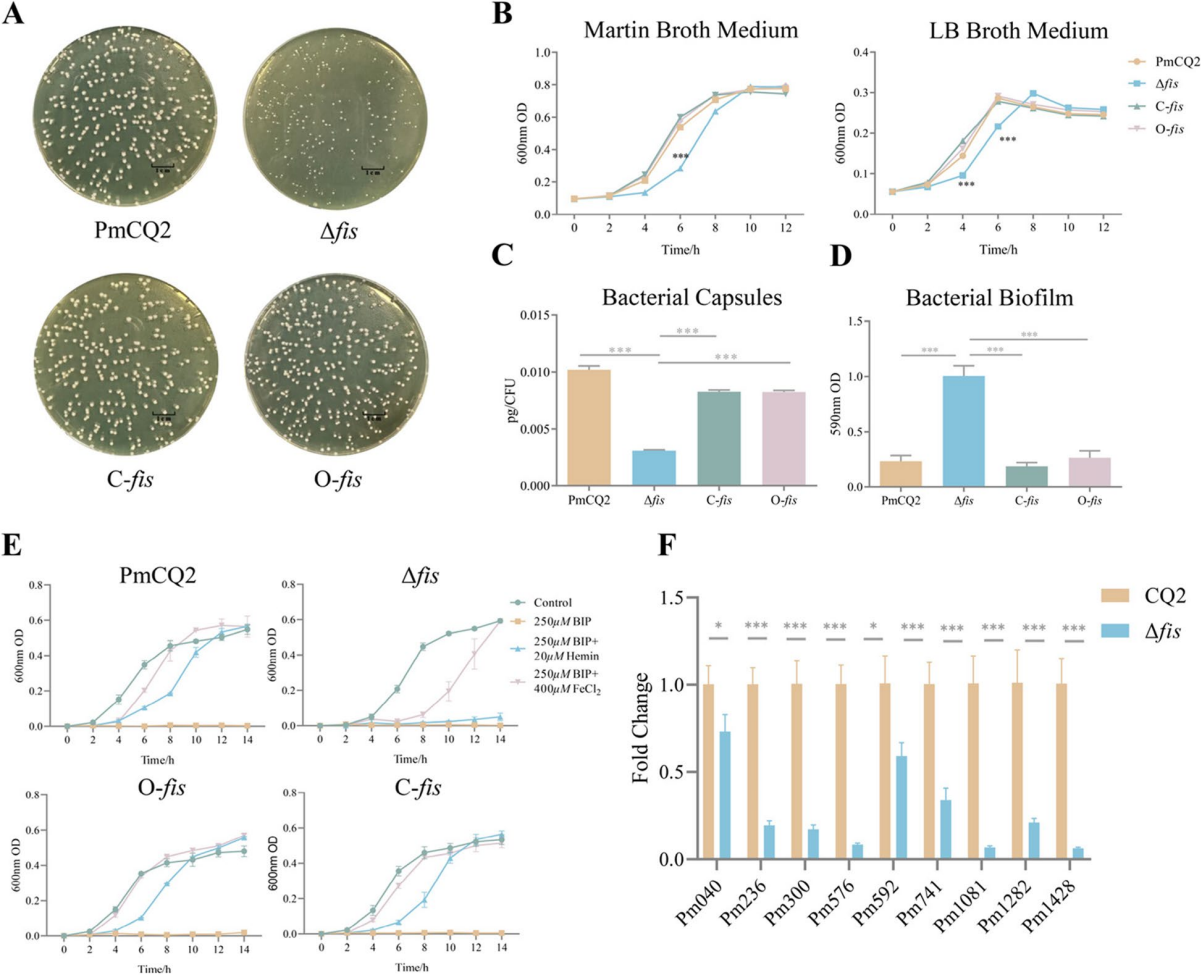
The characteristics of Δ*fis*. **A** Colony morphology of PmCQ2, Δ*fis*, C-*fis* and O-*fis* cultured on Martin agar plates. **B** Growth curves of strains cultured in Martin broth and LB media at 37 °C with shaking at 200 rpm (The experiment was performed in triplicate). **C** Capsular polysaccharide production. **D** Quantification of biofilm production. **E** Heme binds iron uptake. **F** The transcription of haemoglobin receptors in PmCQ2 and Δ*fis*, respectively. All data are expressed as mean ± SD. * *P* < 0.05; ** *P* < 0.01; *** *P* < 0.001; ns means no significant

Compared with the wild-type strain PmCQ2, the *fis* deletion strain has smaller colonies at the same time of growth (Fig. 2A). In Martin broth medium, the wild-type strain PmCQ2 showed a typical growth curve, with a short lag phase (0–2 h), followed by a log phase during which major bacterial growth occurred (2–10 h) and then a stationary phase (10–12 h). In contrast, Δ*fis* grew more slowly at 0–4 h, but the growth rate was reversed at 4–10 h. The OD value of Δ*fis* were significantly lower than those of the wild-type strain between 4 and 8 h. The OD value of complementary strain C-*fis* and overexpressed strain O-*fis* were not significantly different form the parental strain (Fig. 2B). In LB broth medium, the logarithmic growth phase of *P. multocida* was between 2 and 6 h and reached peak in the 6th hour. But Δ*fis* reached peak at the 8th hour and then entered a plateau phase (Fig. 2B). The Δ*fis* has significantly reduced capsule content compared with PmCQ2, C-*fis* and O-*fis* (Fig. 2C). However, the biofilm content of Δ*fis* was significantly higher than other strains (Fig. 2D). Furthermore, in the uptake assay for heme, Δ*fis* was unable to uptake the additional bound iron added to the medium (Fig. 2E). According to report of Bosch et al. on haemoglobin receptors of *P. multocida*, we performed the analysis by RT-qPCR. Transcription of multiple binding haemoglobin receptors of Δ*fis* was inhibited (Fig. 2F). The above results indicate that *fis* gene not only affect bacterial growth but also manipulated capsules biosynthesis. Importantly, Fis manipulate the uptake of bound iron by regulating the transcription of haemoglobin receptors.

### Pathogenicity analyses

**Fig. 3:**
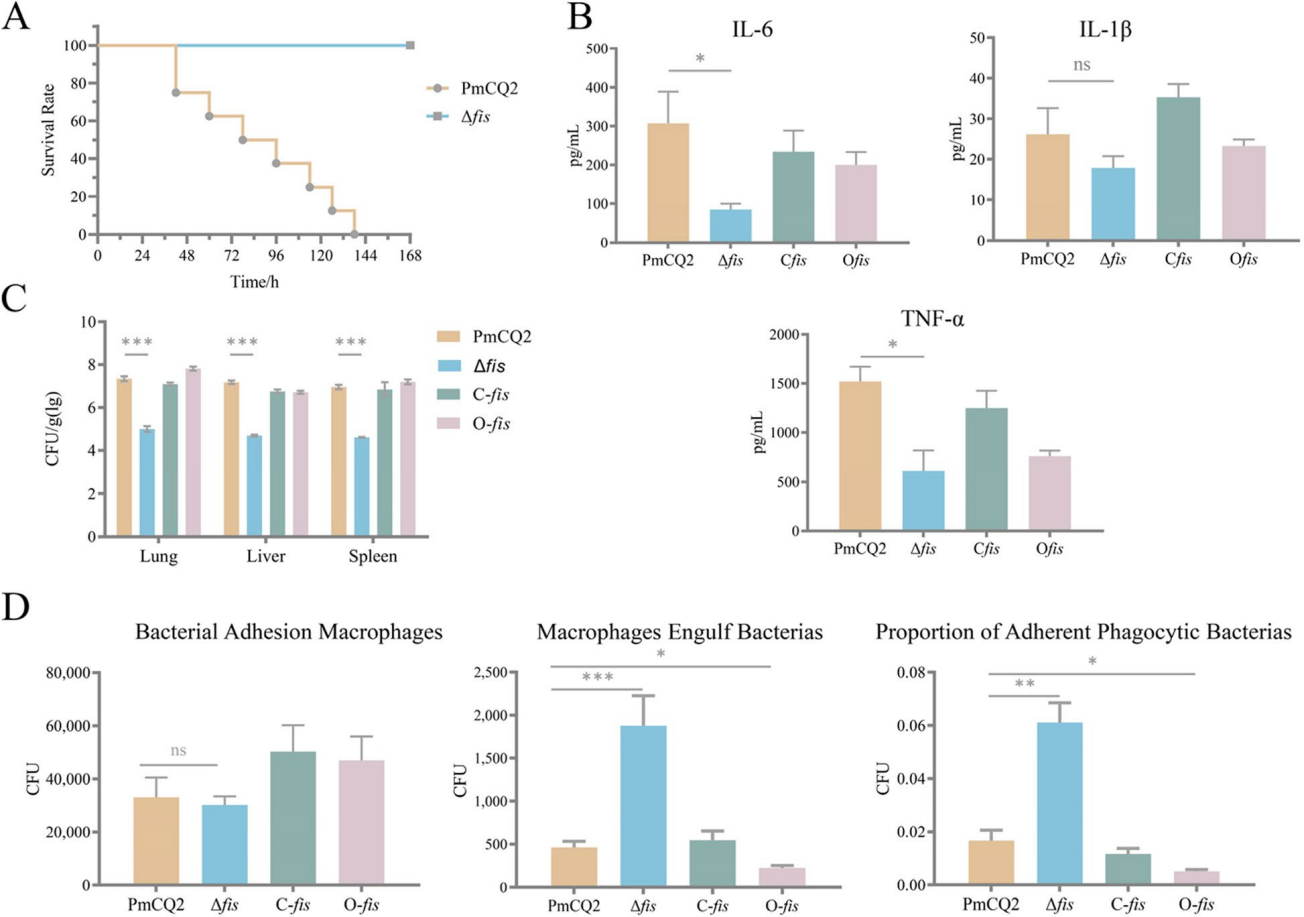
Pathogenicity analyses. A: Survival rates of mice nasal drop infected with PmCQ2 and Δ*fis*, respectively, *n* = 8 mice/group. B: Concentrations of IL-1β, IL-6, and TNF-α in the lung of mice after infection. C: Bacterial loads of lungs, livers, and spleens with PmCQ2, Δ*fis*, C-*fis* and O-*fis* in mice. D: Macrophage engulfment of bacteria and proportion of adherent phagocytic bacteria. All data are expressed as mean ± SD. * *P* < 0.05; ** *P* < 0.01; *** *P* < 0.001; ns means no significant

A series of experiments confirmed changes in the virulence of Δ*fis*. Mice were inoculated nasal drops with PmCQ2 and Δ*fis* (4.0 × 10^5^ CFU), respectively. The survival rate of mice infected with Δ*fis* was significantly higher than those mice infected with PmCQ2 (Fig. 3A). To quantify the decrease of virulence in Δ*fis*, 50% lethal dose (LD_50_) assays were conducted. The LD_50_ of Δ*fis* via intraperitoneal route was 2.37 × 10^8^ CFU, which was 2.37 × 10^8^ fold higher than that of PmCQ2 (LD_50_ = 1 CFU) (Table 1). To further investigate whether the deletion of *fis* attenuates inflammatory cytokines levels and organs bacterial loads, a series of infection experiments was conducted. Compared to those mice infected with PmCQ2, the levels of TNF-α and IL-6 in the lung of Δ*fis* infected mice were significantly lower, while C-*fis* and O-*fis* infected groups showed no significant changes. Additionally, there was no significant change in IL-1β levels between PmCQ2 and Δ*fis* infection (Fig. 3B). The bacterial loads in the lungs of mice infected with Δ*fis* were significantly lower than those infected with PmCQ2 and C-*fis* and O-*fis* (Fig. 3C). In vitro, bacterial resistance to adherent and phagocytosis by mouse macrophages was explored. The results indicated that the deletion of *fis* did not decrease anti-macrophage adhesion to *P. multocida*; however, there was a significant reduction in anti-macrophage phagocytosis (Fig. 3D).

**Table 1:** Determination of the LD_50_ in Δ*fis*

CFU	Mice	Death	Survive	death rate (%)
2 × 10^9^	8	8	0	100
1 × 10^9^	8	8	0	100
5 × 10^8^	8	6	2	75
2.5 × 10^8^	8	4	4	50
1.25 × 10^8^	8	0	8	0

### Cross-protection against *P. multocida* serotypes induced by live Δ*fis*

**Fig. 4:**
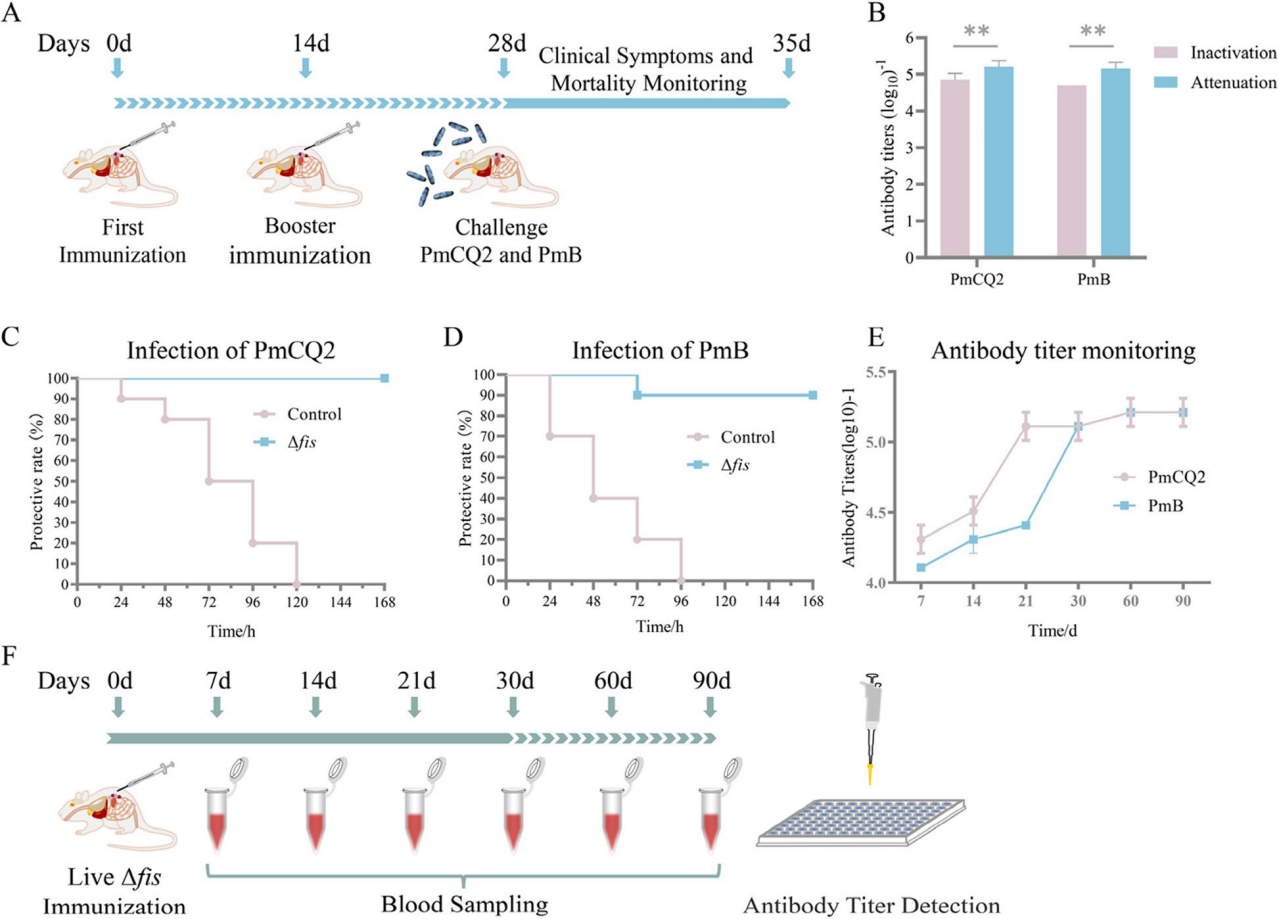
Cross-protection against *P. multocida* serotypes induced by live Δ*fis*. Scheme of immunization and infection **A**. Serum IgG antibody titers **B**. The survival rates of mice subcutaneously immunized with live Δ*fis* and PBS emulsifier and challenged with PmCQ2 (**C**) and PmB (**D**), respectively. Serum IgG antibody titers of PmCQ2 and PmB were monitored 7–90 days after immunization with live Δ*fis* **E**. Scheme of immunization and antibody titer monitoring **F**. All data are expressed as mean ± SD. * *P* < 0.05; ** *P* < 0.01; *** *P* < 0.001; ns means no significant

To explore the effect of *fis* deletion on antibody, the serum IgG titers of the mice immunized with inactivated Δ*fis* and live Δ*fis* were measured by using ELISA. Scheme of immunization and infection was performed as described in Fig. 4A. The antibody titers of live Δ*fis* were significantly higher than inactivated Δ*fis* (Fig. 4B). Both inactivated Δ*fis* and live Δ*fis* provided 100% protection against PmCQ2 (Supplementary Fig. 2 Left A and Fig. 4C). Only live Δ*fis* induced robust immune protection against PmB (90%), whereas inactivated Δ*fis* showed a lower level of protection (30%) (Fig. 4D and Supplementary Fig. 2 Right). Then, mice were immunized with live Δ*fis*, and the monitoring procedure for serum IgG antibody titers was performed according to Fig. 4F. The serum IgG antibody titers against PmCQ2 (serotype A) and PmB (serotype B) peaked at high levels 30 days after immunization with live Δ*fis* and remained stable until 90 days after the conclusion of the study, indicating that live Δ*fis* consistently stimulated the host to secrete sufficient antibodies to against *P. multocida*. (Fig. 4E).

## Discussion

Transcription factors (TF) are essential in regulating bacterial gene expression. As one of TF [25], Fis can enhance the synthesis of transfer and ribosomal RNAs. Interestingly, Fis is ubiquitous, but the function varies among bacteria [25]. In *P. multocida*, Fis regulates bacterial capsule synthesis [8]. Capsule is an important virulence factor of *P. multocida* [26]. The synthesis and transport capsule for *P. multocida* require a variety of proteins to act synergistically [5, 6]. In this study, we observed that deletion of *fis* gene in *P. multocida* resulted in smaller bacterial colonies, lower growth rate, lower capsule and higher biofilm than the wild strain. These results further confirmed that *fis* gene manipulated the growth and capsule synthesis of *P. multocida*. The transcription of the *fis* directly affects the virulence of *P*. *multocida*, as indicated by the survival rate of mice infected with Δ*fis* (Fig. 3A).

Previously, transcriptomic analysis of *fis* natural mutant L28S of *P. multocida* was reported [8]. Thirty-one genes were identified as significantly downregulated and eleven genes as significantly up-regulated in the *fis* mutant strain. Among them, several capsule-associated genes, iron regulatory genes, cross-serotype protective proteins encoded genes, and other virulence genes were downregulated. However, the Fis protein is predicted to contain 4 functional domains, single point mutations may not be sufficient to characterize the function of the whole *fis*, therefore we deleted the whole *fis* from the genome for more precise functional investigation. Our results also showed that the *fis* deletion strain utilized free iron ions in Martin broth medium, but was unable to utilize bound heme directly and showed a significant reduction in virulence. These results are consistent with our previous results [27] and the transcriptome results of Steen et al. [8]. In addition to lspB 2 (Hemolysin accessory protein), Pm1078 (Hemin binding receptor) and exbB (Iron regulated virulence protein) mentioned in the results of Steen et al. [8] We also found that Fis manipulate the uptake of bound iron by other regulating the transcription of haemoglobin receptors, including Pm040, Pm236, Pm300, Pm576, Pm592, Pm741, Pm1081, Pm1282 and Pm1428. In subsequent experiments, we expressed Fis with Myc-tag in the wild strains PmCQ2 to attempt to pull down the related proteins, but were unsuccessful, and the specific molecular mechanism still needs to be further explored.

To clarify the contribution of *fis* to the virulence of *P. multocida*, a *fis* mutant strain was evaluated in mice. In our previous work, we found that inflammatory storm contributed to Pm-induced host death [20, 21]. Here, mice infected with *fis* deletion strain showed significantly lower TNF-α and IL-6 expression in the lung compared to mice infected with wild type strain, indicating *fis* deletion reduced host inflammatory reactions. Interestingly, the IL-1β secretion was not reduced by the absence of the *fis* gene. This result suggested that IL-1β secretion was not only mediated by bacterial capsule. Moreover, mice infected with *fis* deletion strain exhibited lower bacterial loads in organs. The *fis* deletion strain was also more phagocytosed by macrophages in vitro. Thus, the *fis* deletion strain tended to be clear by the host immune system. In summary, the *fis* deletion strain exhibited less virulence and a milder inflammatory response in mice.

*Pasteurella* types A and B are the most harmful types to the beef cattle industry, and it is particularly important to develop cross-protection vaccines. Some commercial vaccines of *P. multocida* protect bovine against infection with homologous serotypes [28], but the lack of a universal vaccine showed strong cross-protection against *P. multocida* infection. In the present study, the live Δ*fis* not only stimulates the high levels of antibody, but also produces immune protection against *P. multocida* type B. The LD_50_ of Δ*fis* was increased by 2.37 × 10^8^ fold, and the virulence reduction was very significant, which be developed as an attenuated vaccine candidate strain. In addition, the general virulence was decreased in Δ*fis*, but the expression of some virulence-related genes such as pm1819 and pm1820, which encode proteins with similarity to the *Salmonella* SrfB and SrfC proteins, were up-regulated [8]. The *fis* deletion strain lacks capsule, which is more likely to be phagocytosed by macrophages to present antigen and moderately stimulate the secretion of inflammatory cytokines. These may all contribute to the ability of Δ*fis* to protect against serotype A and B. Although the antibody titer against *Pasteurella* type B was high in mice immunized with inactivated Δ*fis*, it did not protect mice from *Pasteurella* type B infection. This is due to the decreased expression of several immune proteins that can stimulate cross-serotype protective immunity against *P. multocida* infection after *fis* deletion, such as PlpE [8]. It is worth mentioning that, in our previous study, one *P. multocida* mutant with a substantial deletion of a DNA fragment encompassing six genes (PmCQ2Δ4555–4580) exhibited notable attenuation of virulence and displayed remarkable cross-protection. The *fis* is one of them [27].

## Conclusion

The *fis* regulated capsule production, biofilm formation, and virulence in *P. multocida*. Our study firstly demonstrated that *fis* manipulated the intake of bound iron ion, by regulating multiple haemoglobin receptors in *P. multocida*, and the *fis* deletion strain can be used as an attenuated vaccine candidate against *P. multocida* of A and B serotypes.

## Methods

### Bacterial strains and growth conditions

The highly virulent bovine *P. multocida* capsular type A CQ2 (PmCQ2, GenBank accession number: No. CP033599) was isolated from a lung of calf with pneumonia in Chongqing, China [29]. Bovine *P. multocida* capsular serotype B strain B (B: L2, PmB) was purchased from the China Institute of Veterinary Drug Control [21]. PmCQ2 and PmB was streaked on Martin agar plate (Qingdao Hope Bio-Technology Co., Ltd., Qingdao, China) and incubated for 24 h at 37°C. Single colony was inoculated into 5 mL Martin broth and cultured for 12 h at 37°C with shaking at 220 rpm.

### Construction of plasmids

To enhance the knockout efficiency of the knockout plasmid (pUC19ori^TS^Kan^R^) [30], pUC19ori^TS^Kan^R^NgAgo was constructed as described by Fu et al. [31]. A *NgAgo* gene (GenBank ID: KU899087.1) and a ribosome binding sequence (rbs) were biosynthesis by BGI (BGI, Shenzhen, China) and then inserted in upstream of the kanamycin resistance gene (Kan^R^) in the pUC19ori^TS^Kan^R^ to generate the pUC19ori^TS^Kan^R^NgAgo plasmid (The seamless cloning kit was provided by Hanbio Biotechnology, China) with the pUC19ori^TS^Kan^R^-F/R and NgAgo-rbs-F/R primers (Supplementary Table 1).

Construction of the knockout plasmid pUC19ori^TS^Kan^R^NgAgo-Δ*fis*. A 335 bp upstream and 334bp downstream homology arm of *fis* gene was amplified by using PmCQ2 genomic DNA as a template with the primers Fis-UF/UR and Fis-DF/DR. The upstream and downstream homology arms were combined into *fis* homology arms by fusion PCR. Next, the PCR product was inserted into the HindIII and BamHI sites of temperature-sensitive knockout plasmid pUC19ori^TS^Kan^R^NgAgo to generate the plasmid pUC19ori^TS^Kan^R^NgAgo-Δ*fis*.

Construction of the overexpress plasmid pUC19ori^TS^Kan^R^-*fis*. Briefly, a 349 bp segment containing initiation codon of *fis* gene, HA and Myc tag was amplified by using PmCQ2 genomic DNA as a template with the primers *fis*-F/R and then replaced NgAgo in upstream of the rbs in the pUC19ori^TS^Kan^R^NgAgo to generate the pUC19ori^TS^Kan^R^-*fis* plasmid with the pUC19ori^TS^Kan^R^-*fis*-F/R primers.

Construction of the knock-in plasmid pUC19ori^TS^Kan^R^-c*fis*. A 1144 bp contained *fis* gene and the upstream and downstream homology arms was amplified by using PmCQ2 genomic DNA as a template with the primers Fis-UF/DR. Next, the PCR product was inserted into the HindIII and BamHI sites of temperature-sensitive knockout plasmid pUC19ori^TS^Kan^R^NgAgo to generate the plasmid pUC19ori^TS^Kan^R^NgAgo-c*fis*. Construction of plasmids are shown in Supplementary Fig. 1A.

### Construction of mutant strain

The knockout/overexpress plasmid and knock-in plasmid were transferred into PmCQ2 and Δ*fis* by electroporation (2500 V, 5 ms), respectively. The strains with plasmids were screened on Martin agar plates with kanamycin (100 μg/mL) at 30 °C and verified by PCR with primers KO-F/R, EO-F/R and Fis-UUF/DDR. Subsequently, 100 μL Δ*fis*, transformants was spread onto Martin agar plates and grown at 42 °C for 16 h to allow plasmid resolution. KMT1-F/R primers were used to identify *P. multocida* [32, 33]. Primers sequences are listed in Supplementary Table 1, strains and plasmids are listed in Supplementary Table 2.

### Real-time-quantitative -PCR (RT-qPCR)

Total RNA of the bacterial samples was extracted by RNA Kit (ABclonal, China) based on the manufacturer’s instructions. Next, extracted RNA was reverse transcribed to cDNA by Reverse Transcription Master Mix (ABclonal, China) according to manufacturer’s recommendations. Then, RT-qPCR was performed via SYBR Green on a CFX96 instrument (Bio-Rad). Relative expression of genes was calculated using the 2^−ΔΔCt^ method with 16S as reference. Primer sequences for RT-qPCR are listed in Supplementary Table 3.

### Western blot

O-*fis* was collected by centrifugation in the logarithmic phase. The pellets were washed 3 times and re-suspended with sterile PBS (pH 7.4). After sonication of the resuspended bacterial suspension, protein loading buffer was added and completely denatured by a boiling water bath for 10 min. Fis proteins were detected by using 15% SDS-PAGE and blotted electrophoretically onto a polyvinylidene difluoride (PVDF) membrane. The membrane was incubated with Myc tag antibody (1:2500; Huabio, China) and horseradish peroxidase-conjugated goat anti-rabbit IgG antibodies (1:20,000; Sigma). An Enhanced Chemiluminescent reagent (Pierce; Thermo) was applied for a chromogenic reaction. Proteins were visualized using a LTQ Mass Spectrometer (Shanghai Applied Protein Technology Co. Ltd., Shanghai, China).

### Determination of bacterial growth curve

Logarithmic bacterial cells (10 μL, 1.0 × 10^7^ CFU) were added into 5 mL fresh sterile Martin broth and LB broth liquid, then incubated at 37 °C with shaking at 220 rpm, and OD_600_ values of the bacterial cultures were determined at every 2 h.

### Heme iron uptake

To explore the effect of Fis proteins on heme uptake, 250 μM bipyridine were used to chelate free iron ions in the Martin broth. Meanwhile, 20 μM heme and 400 μM ferrous chloride were added to Martin broth treated with bipyridine. PmCQ2, Δ*fis*, C-*fis*, and O-*fis* was inoculated into treated Martin broth and cultured for 12 h at 37°C with shaking at 220 rpm. Samples were measured optical density at 600 nm (OD_600_) every 2 h to plot the growth curve. According to report of Bosch et al. on haemoglobin receptors [34] of *P. multocida*, we performed the analysis by RT-qPCR.

### Quantification of hyaluronic acid in the capsule of bacteria

Bacteria were grown in 100 mL Martin broth and incubated at 37 °C with 220 rpm for 8 h to logarithmic phase. The supernatant was removed after centrifugation at 7600 g for 10 min, washed twice with PBS and resuspended. Next, incubation for 1h at 42 °C, the centrifuged supernatant was used to determine the capsule content. To be specific, after the capsule staining solution (0.2 mg/mL Stain all, 0.06% glacial acetic acid in 50% formamide) was mixed with the sample or standard substance at a ratio of 1: 9, the absorbance of OD_630_ was measured by a microplate reader. The standard curve was drawn by setting the standard substance gradient and calculate the capsule content.

### Biofilm assay

Biofilm assay was conducted based on the method described by Petruzzi et al. [35], 100ul (1.0 × 10^7^ CFU) of bacteria were inoculated in 96-well cell culture plates and incubated for 48 h at 37 ℃. The bacterial cultures of each well were drawn off, methanol (100 μL/well) was added into each well and fixed for 30 min, washed with PBS for 3 times and dried for 1 h. Then 0.1% crystal violet staining solution (100 μL/well) was added into each well and stained at 37 °C for 15 min, discarded crystal violet solution in each well and washed with PBS for 3 times and dried at room temperature. Finally, 33% glacial acetic acid solution was added and measured at OD_590_ by microplate reader.

### Mice and ethics statement

Kunming mice (Female, 25–30 g) were purchased from the SPF (Sibeifu Biotechnology Co., Ltd., Beijing) and housed in individually ventilated pathogen-free cages (temperature at 20–30 °C, relative humidity at 50–60%, and lighting cycle at 12 h/day) with free access to food and water. The mice were acclimatized for 4 days after arrival to the laboratory before starting the experiments. All animal experiments were carried out with approval from the Animal Ethics and Research Committee of Southwest University (SWU_LAC2023120053), Chongqing, China. After finishing the animal experiments, the mice were anaesthetized with 1.5% pentobarbital sodium and then euthanized for blood and tissue collection.

### Pathogenicity of mutant strain

To determine the pathogenicity of the PmCQ2 and Δ*fis*. Kunming mice (*n* = 8) were infected by nasal drop infection with PmCQ2 and Δ*fis* at a dose of 4 × 10^5^ CFU in 10 μL, and the numbers of mice used for each strain detection were equal. Mice were monitored for 7 days to determine the survival curves, mice showing severe clinical signs (e.g., depression, accelerated breath, cough, hairiness, and lethargy) were considered moribund and were humanely killed.

### Fifty (50%) lethal dose LD_50_ measurements in mice

To determine the impact of Fis protein on PmCQ2 virulence, 100 female KM mice (7/8-week-old) were randomly divided into 8 groups (*n* = 8). Δ*fis* groups (5 groups) of mice were infected intraperitoneal with 100 μL of various doses Δ*fis* (1.25 × 10^8^, 2.5 × 10^8^, 5.0 × 10^8^, 1.0 × 10^9^, and 2.0 × 10^9^ CFU), respectively. Mice were monitored for 7 days to determine the survival curves, and mice were humanely euthanized with severe clinical signs. Then, the numbers of survived mice in each group were recorded, and the Lethal Dose 50% (LD50) was calculated with the method of Bliss [36].

### Quantitation of *P. multocida* associated with macrophage

Peritoneal macrophages were isolated from mice as previously described [20, 20]. Peritoneal macrophages were cultured in RPMI 1640 medium with 10% heat-inactivated fetal bovine serum and counted with a hemocytometer, and then incubated overnight at 37 °C with 5% CO2 in 48-well microplates at a density of 1.0 × 10^6^ cells/well. The macrophages challenged with *P. multocida* at a multiplicity of infection (MOI) of 1 for 6 h. Cells were washed three times with chilled PBS (Total number of adhered and phagocytosed *P. multocida*), and the cells of another 48-well microplate were treated with 1% penicillin–streptomycin for 30 min and washed 3 times with PBS (number of phagocytosed *P. multocida*). The cell lysates were diluted with PBS and grown on Martin’s agar plates at 37°C for 24 h to determine the number of *P. multocida*. The number of adhered *P. multocida* was equal to the total number of adhered and phagocytosed *P. multocida* minus the number of phagocytosed *P. multocida*.

### Bacterial colonization and lung inflammatory factor detection

Mice (*n* = 4 / group) were intraperitoneally infected with 2 × 10^5^ CFU of PmCQ2, Δ*fis*, C-*fis*, and O-*fis* strains for 14 h, respectively. And mice were euthanized for collection of lung, liver and spleen tissues to measure the bacterial loads (*n* = 4 / group) [37]. The lung inflammatory cytokines (TNF-α, IL-1β and IL-6) were detected by ELISA kits (Invitrogen, USA).

### Immunization and challenge trial

To determine immune protection of live Δ*fis* and inactivated Δ*fis*, 80 female KM mice were randomly divided into 8 groups (*n* = 10/group). Mice were subcutaneous injected with inactivated Δ*fis* (2 groups), PBS emulsifier (2 groups), live Δ*fis* (2 groups), and PBS (2 groups), respectively. Mice received booster immunization 14 days after the first immunization. Fourteen days later, mice were intramuscularly infected with PmCQ2 (2.0 × 10^7^ CFU, 4 groups) and PmB (1.0 × 10^7^ CFU, 4 groups). The specific operation and dose of immunity and infection is shown in the Fig. 4A and Table 2. The mice were monitored for 7 days after challenge, which showing severe clinical signs were considered moribund and were humanely euthanized, and the numbers of survived mice in each group were recorded.

**Table 2:** Scheme of immunization and infection

Groups	Immunization	First Immunization (0 d)	Booster Immunization (14 d)	Strain of Challenge (28 d)
1	Inactivated Δ*fis*	1.0 × 10^9^ CFU	5.0 × 10^8^ CFU	PmCQ2
2	Inactivated Δ*fis*	1.0 × 10^9^ CFU	5.0 × 10^8^ CFU	PmB
3	PBS emulsifier	200 μL	100 μL	PmCQ2
4	PBS emulsifier	200 μL	100 μL	PmB
5	Live Δ*fis*	3.0 × 10^8^ CFU	4.0 × 10^8^ CFU	PmCQ2
6	Live Δ*fis*	3.0 × 10^8^ CFU	4.0 × 10^8^ CFU	PmB
7	PBS	200 μL	100 μL	PmCQ2
8	PBS	200 μL	100 μL	PmB

### Statistical analysis

Data about characteristics of Δ*fis* are expressed as the mean ± standard deviation (SD) from three independent experiments (*n* = 3), and data about pathogenicity analyses are expressed as the mean ± standard deviation (SD) from four independent experiments (*n* = 4). All statistical analyses were performed using GraphPad Prism software. The survival rates of the mice were evaluated using Kaplan‒Meier analysis (Prism 6.0). All the other data between two groups were evaluated using unpaired, two-tailed Student’s *t* test (Prism 6.0). Significant differences were considered as *p* < 0.05 (∗ *p* < 0.05, ∗ ∗ *p* < 0.01, ∗ ∗ ∗ *p* < 0.001).

## Supplementary Information

Supplementary Material 1.

Supplementary Material 2.

## Data Availability

The data used to support the findings of this study are available from the corresponding author upon reasonable request.

## Abbreviations

Pm: Pasteurella multocida
LD50: Median lethal dose
CFU: Colony-forming units
PCR: Polymerase chain reaction
RT-qPCR: Real-time-quantitative-PCR
PBS: Phosphate buffer saline
TNF-α: Tumor necrosis factor α
IL-6: Interleukin-6
IL-1β: Interleukin-1 β
