# Supplementary material for: Deleting fis downregulates virulence and effectively protects Pasteurella multocida infection in mice
Source: BMC Vet Res. 2025 May 7;21:323. doi: 10.1186/s12917-025-04769-x (PMC12057170; doi:10.1186/s12917-025-04769-x)
Supplement: Supplementary file 1 — Supplementary Material 1. [file 12917_2025_4769_MOESM1_ESM.docx]

Supplementary Table 1 Primers of mutant construction and protein expression used in this work.

| Primer name | Sequence (5’-3’) | Product (bp) | Reference |
| --- | --- | --- | --- |
| pUC19ori^TS^Kan^R^-F | ATGAGCCATATTCAACGGGAAACGTCTTGC | 5096 | This study |
| pUC19ori^TS^Kan^R^-R | ACGTCGTATGGGTACATGTAATTTTCCTCCTATCAACA |  | This study |
| NgAgo-rbs-F | ATGTACCCATACGACGTCCCAGACTACGCTACAGTGATTGACCTCGATTCGA | 2849 | This study |
| NgAgo-rbs-R | CCGTTGAATATGGCTCATTATGCACTCCTATTATTTAACTAAG |  | This study |
| Fis-UF | ATTTATCGGTACCCGGGGATCCCCGAGTTATGGAAAGCTT | 335 | This study |
| Fis-UR | TACAATGGTCCGGGATTACGTTGTTGTTCTAAC |  | This study |
| Fis-DF | ACAACGTAATCCCGGACCATTGTAGTATTTAAAC | 334 | This study |
| Fis-DR | GACCATGATTACGCCAAGCTTGCTTTTTGAAAACGCACA |  | This study |
| Fis-UUF | GGACGTGGTTCATTAGGCAAT | 972/695 | This study |
| Fis-DDR | GGGTTCAGATAAGTCAGCAAAATGA |  | This study |
| *fis*-F | ATACGACGTCCCAGACTACGCTTTAGAACAACAACGTAATCCTGCTG | 349 | This study |
| *fis*-R | TTACAGATCCTCTTCAGAGATGAGTTTCTGCTCACCCATACCGTACTTTTTTAATTTC |  | This study |
| pUC19ori^TS^Kan^R^-*fis*-F | CATCTCTGAAGAGGATCTGTAATTGATATTTTTTCTTGCAAAGAACCAGTTG | 5271 | This study |
| pUC19ori^TS^Kan^R^-*fis*-R | AGCGTAGTCTGGGACGTCGTATG |  | This study |
| KO-F | CGCAACGCAATTAATGTGAG | 867/289 | This study |
| KO-R | GTTCCCACTATCAAATTGACAG |  | This study |
| OE-F | ATACGACGTCCCAGACTACGCTTTAGAACAACAACGTAATCCTGCTG | 516 | This study |
| OE-R | TTACAGATCCTCTTCAGAGATGAGTTTCTGCTCACCCATACCGTACTTTTTTAATTTC |  | This study |

Supplementary Table 2 Bacterial strains and plasmids used in this study.

| Plasmids or strains | Description | Source |
| --- | --- | --- |
| **Plasmids** |  |  |
| pUC19ori^TS^Kan^R^ | Basic plasmids for mutation and complement construction. | Lab collection |
| pUC19ori^TS^Kan^R^NgAgo | Insertion of NgAgo gene into pUC19. | This work |
| pUC19ori^TS^Kan^R^NgAgo-Δ*fis* | For the deletion of *fis* in PmCQ2. | This work |
| pUC19ori^TS^Kan^R^-*fis* | For the overexpression of fis in PmCQ2. | This work |
| pUC19ori^TS^Kan^R^NgAgo-c*fis* | For the knock in of *fis* in PmCQ2-Δ*fis.* | This work |
| pET-30a | Basic plasmid for protein expression. | Lab collection |
| pET-30a-Fis | For the expression of *P. multocida* Fis. | This work |
| **Strains** |  |  |
| PmCQ2 | Wild-type and virulent *P multocida.* | Lab collection |
| Δ-*fis* | *fis* gene deleted strain in PmCQ2. | This work |
| O-*fis* | *fis* gene overexpress strain in PmCQ2. | This work |
| C-*fis* | *fis* gene complementary strain in PmCQ2-Δ*fis.* | This work |
| *E. coli* DH5α | For storing plasmids | Lab collection |

Supplementary Table 3 Primers of RT-qPCR used in this work.

| Primer name | Sequence (5’-3’) | Product (bp) | Reference |
| --- | --- | --- | --- |
| Q-*fis*-F | ATTGTCGCTGCACGAGTTTG | 122 | This study |
| Q-*fis*-R | TCGCAGTTAGATGGCCAAGA |  |  |
| Pm040-F | CGACACAGCAACAATGGCAA | 193 | This study |
| Pm040-R | ACACGCAGACCGTAACCAAT |  |  |
| Pm236-F | ACGACGGGCATTTGGGTTAT | 78 | This study |
| Pm236-R | GCCGGGTTTGAAAATGGCTT |  |  |
| Pm300-F | CCGTTGAGTAGCCCGCTTTA | 135 | This study |
| Pm300-R | TCGCGAAAGGTGCTGATTCT |  |  |
| Pm576-F | GCGCCGTCTAGTTGAACTCT | 98 | This study |
| Pm576-R | GGGGTAAATATGGCGGGAGG |  |  |
| Pm592-F | GACCGATATCGAACCTGCGT | 125 | This study |
| Pm592-R | ATTCACGGCTCGGCTGATAG |  |  |
| Pm741-F | GGGTGAGATCAAGCAAGCCT | 106 | This study |
| Pm741-R | GCGTATGGCACGTGGAAATC |  |  |
| Pm1081-F | TGTGCGATAATCACCGGCTT | 108 | This study |
| Pm1081-R | AGTGCGGACAATGAACGTCT |  |  |
| Pm1282-F | TTCTCGTGGTCAAGGTGGTG | 142 | This study |
| Pm1282-R | ATTAACTTCGGGTGCCCGTT |  |  |
| Pm1428-F | ACGGTACGTTGGCGGTAAAT | 105 | This study |
| Pm1428-R | GGTGTGGAAGGGCGTATTGA |  |  |

A


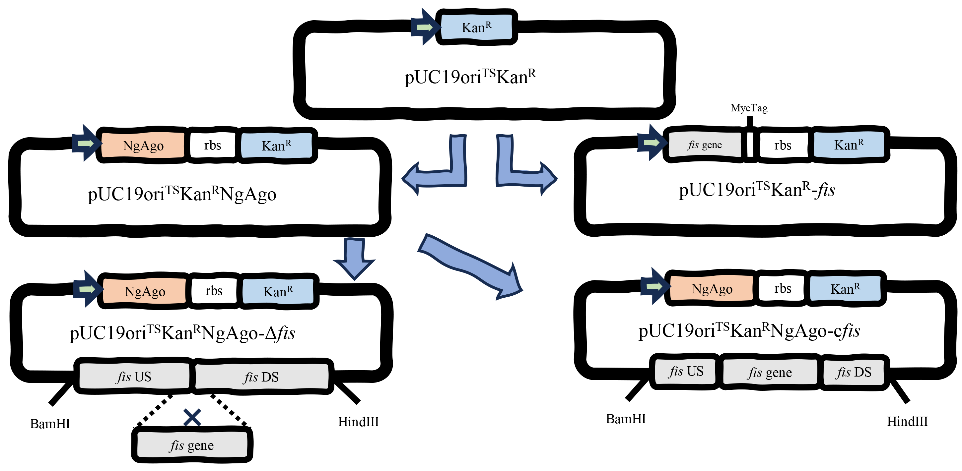


B


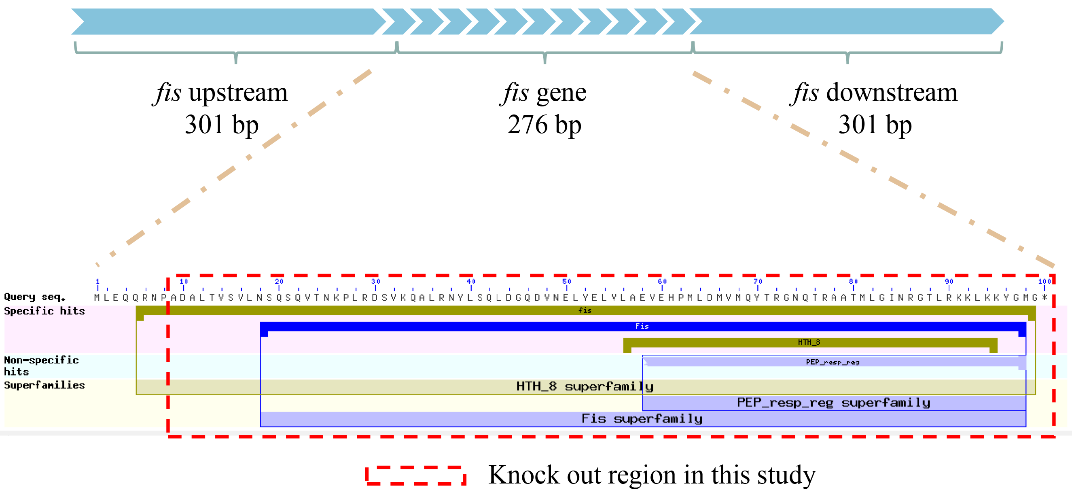


Supplementary Figure 1. Construction of the *fis* deletion strain (Δ*fis*), complementary strain (C-*fis*) and overexpress strain (O-*fis*). A: Schematic representation of plasmids construction. B: Schematic representation of the construction of fis deletion strain.


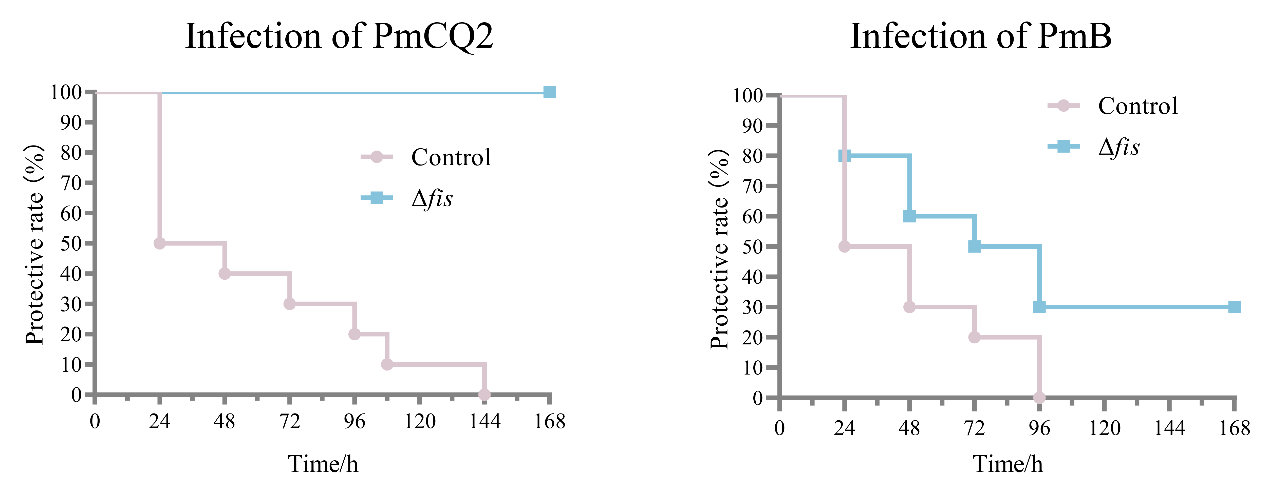


Supplementary Figure 2. The survival rates of mice subcutaneously immunized with inactivated Δ*fis* and PBS emulsifier and challenged with PmCQ2 (Left) and PmB (Right)
