# Supplementary figures and images for: Deleting fis downregulates virulence and effectively protects Pasteurella multocida infection in mice
Source: BMC Vet Res. 2025 May 7;21:323. doi: 10.1186/s12917-025-04769-x (PMC12057170; doi:10.1186/s12917-025-04769-x)

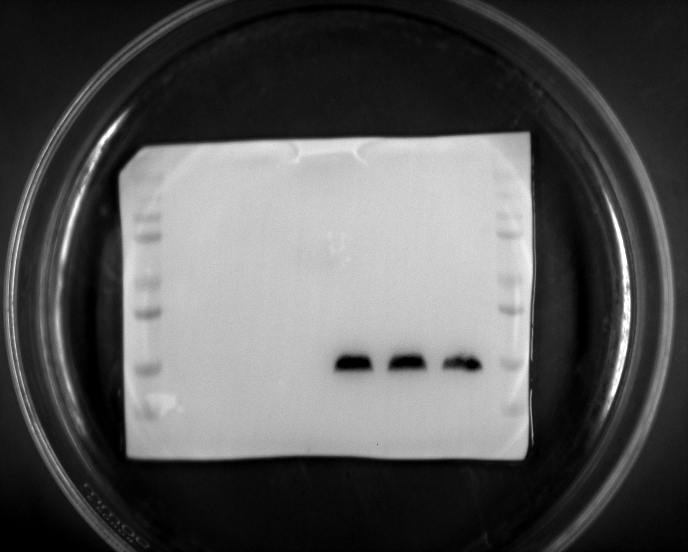

Supplement: Supplementary file 2 — Supplementary Material 2. [file 12917_2025_4769_MOESM2_ESM.zip › Fis-Myc-tag.tif]

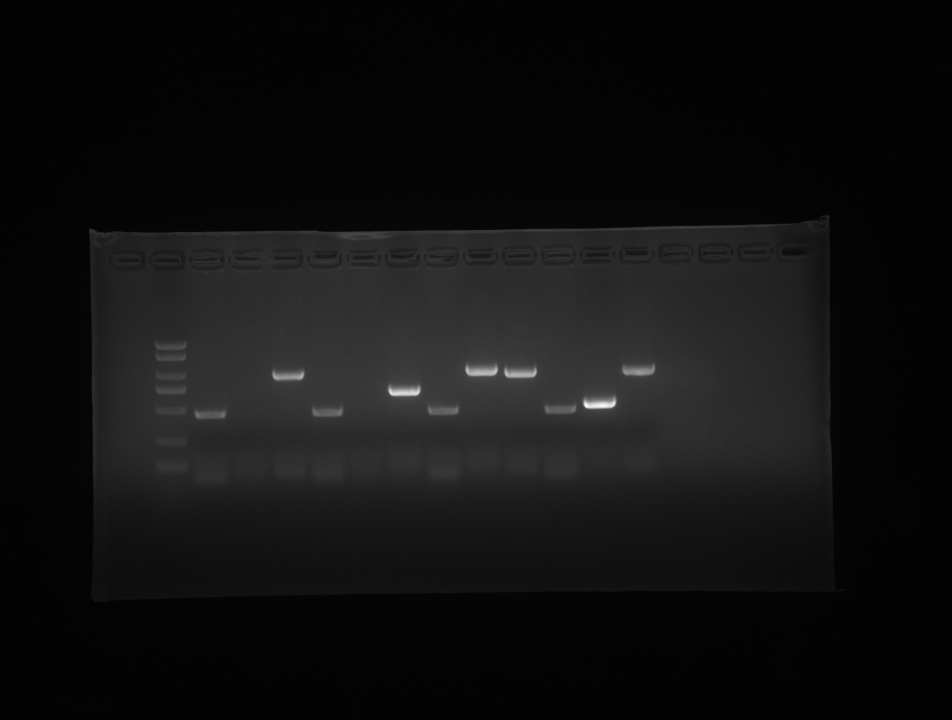

Supplement: Supplementary file 2 — Supplementary Material 2. [file 12917_2025_4769_MOESM2_ESM.zip › PCR confirmation of PmCQ2, fis, C-fis and O-fis.tif]

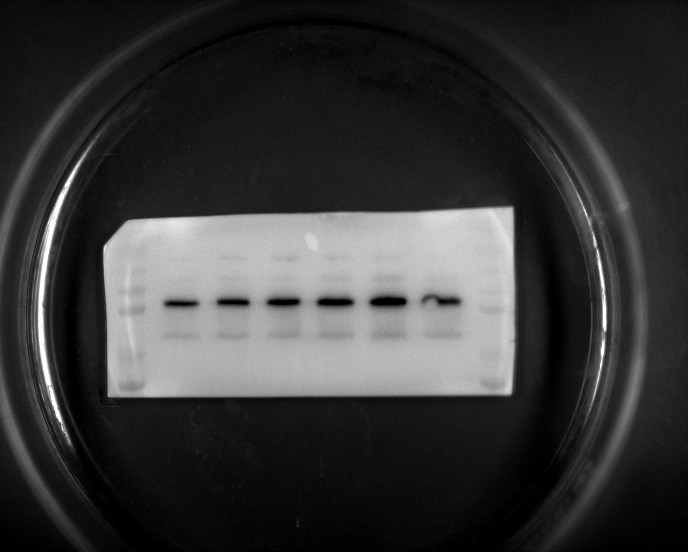

Supplement: Supplementary file 2 — Supplementary Material 2. [file 12917_2025_4769_MOESM2_ESM.zip › PmCQ2 RS09000.tif]
